# The Rolling 50s (and More): Cars and Life Satisfaction Among Seniors Across Europe

**DOI:** 10.1007/s11482-020-09887-2

**Published:** 2020-11-24

**Authors:** Gaël Brulé, Laura Ravazzini, Christian Suter

**Affiliations:** University of Neuchâtel, 27 faubourg de l’hôpital, 2000 Neuchatel, Switzerland

**Keywords:** Wealth, Car, SWB, Elderly, Sustainable development, Materialistic values

## Abstract

Cars represent a valuable real asset that most individuals use on a daily basis. Although cars are a form of material prosperity like income and other forms of wealth, the link between cars and subjective well-being (SWB) is barely covered in the existing literature. Furthermore, few existing contributions are scattered across specific cultural contexts. Here, we analyze the relationship between cars and the SWB of seniors in different European countries using the SHARE dataset. We construct multilevel and fixed-effect models to explore the extent of economic, infrastructural, and cultural factors and how they can explain this relationship. The results show that the value of the car is, among all wealth components (houses, bank account, bonds, stocks, mutual funds, debts and mortgages), the form of wealth most related to life satisfaction. In addition, cars matter less (a) in affluent societies, (b) where rail infrastructure is more developed, and (c) where people hold fewer materialistic values. We discuss these results in the framework of the functional and positional value of cars, i.e., respectively, the value derived from it regardless of others and the value derived from it vis-à-vis others.

## Introduction

Material prosperity has been evaluated through income although wealth represents a better proxy (Skopek 2014). This is particularly true for seniors who have generally little income and who have accumulated wealth over several decades. Wealth depicts individuals’ long-term consumption potentials and capacities to maintain a good standard of living (Spilerman 2000; Hochman and Skopek 2013; Brulé and Suter 2019). On top of providing income (e.g., through investment), wealth also provides utility (e.g., by increasing personal freedom), security (e.g., in case of unemployment), socialization (e.g., when raising kids), power (e.g., political), inheritance, and status. Their relative importance depends on the type of asset: While financial assets can provide stability for the current and the future generations, real assets are visible indicators of social status and can be used on a daily basis for different purposes (e.g., work, leisure activities, and socialization).

Cars are the second-most important type of spending among real assets after the house (Okulicz-Kozaryn et al. 2015; Urry 2004). Some households use loans and reduce their consumption to buy their car. Cars not only crystallize financial considerations but they also reveal socio-cultural representations because they are deeply embedded in the social life (Conley and McLaren 2016). They convey social status for individuals or families by showing their purchasing power on the road and on the parking lot (Litman and McLaren 2016). This is because vehicles carry a symbolic value in the popular culture by which cars communicate one’s identity. Cars are linked to the way people are perceived and the way people perceive themselves. Expensive cars are status symbols. The literature on conspicuous consumption enumerates cars in the goods of high symbolic content such as houses and jewels (Corneo and Jeanne 1997; Heffetz 2011; Winkelmann 2012). In addition, showing the value of the car has an influence on one’s social environment. Kuhn et al. (2010) demonstrate that living next to a winner of the Dutch Postcode Lottery increases the level of car consumption in the neighborhood.

There are several factors that can describe the reasons why individuals would enjoy a certain good. Hirsch (1976) distinguishes the *functional* from the *positional* value to separate the enjoyment provided by the function of the item and the differentiating effect it can embody. Within the specific case of cars, one can define the former as the utility people derive from using a “driving unit” and the latter as the benefits people gain by showing it in terms of status. The functional value of cars is what enables one to move from one place to another; the positional value is anything that would come on top. The functional value is important for individual well-being insofar as it enables individuals to move to work, socialize, and travel. In that sense, it creates opportunities for individuals to achieve higher well-being for individuals. In terms of positional value, previous studies have shown that the positional value is particularly important in the case of cars (see e.g., Steg 2005). Functional and positional values correspond to an absolute and a relative value, both matter from the perspective of SWB (Diener et al. 1993). The relationship between the functional (absolute) value of cars and SWB depends on the satisfaction derived from being able to move from point A to point B. The relationship with its positional (relative) value depends on social scarcity; more precisely, it depends on the satisfaction derived from being part of a group of a few people with a rare object such as an expensive car. Satisfaction is derived from positional goods and is highly dependent on social comparisons (Solnick and Hemenway 1998). As stated by Hirsch (1976, p.2), “the satisfaction that individuals derive from goods and services depends in increasing measure not only on their own consumption but on consumption by others as well.” This implies that households owning a car can benefit from the functional value of the car.

There are several reasons why studying these two types of car values (and in particular its positional value) might be particularly interesting for seniors. First, seniors are less likely to have received their car as a gift and more likely to have acquired it via their own means (Kotlikoff 1988). Second, among the elderly, cars are a visible sign of the status achieved during their past professional activity that they cannot easily show otherwise. Therefore, the value of their cars is quite likely to reflect their own choices. Third, older generations usually have more materialistic values than younger generations (Inglehart 2008; Jaspers and Pieters 2016) who are more interested in the sharing economy than in the possession of cars as status symbols (Capus 2019). Thus, older generations might be more willing to spend on cars than on travel or other intangible activities.

The links between cars and well-being, whether psychological or subjective, have been investigated mostly from the perspective of access to public transportation or car ownership (see e.g. Morris and Guerra 2015). However, this usually tackles the functional value—the positional value is typically absent. Only in a few cases has the value of the car been investigated from the perspective of subjective well-being (SWB). Okulicz-Kozaryn et al. (2015) showed that there are no significant differences between frugal car owners and expensive car owners in terms of SWB in the USA. Winkelmann (2012) found that the prevalence of Ferraris and Porsches in Swiss municipalities has a negative impact on income satisfaction, but this cannot be generalized to life satisfaction. Still, these studies concern specific car brands and/or specific socio-cultural contexts.

There could be several reasons why the relationship between cars and SWB might be dependent on the national context. Economic, infrastructural, and cultural factors might influence the relationship between cars and SWB. First, the level of affluence of a country could favor or inhibit this relationship through two possible routes, i.e., either by improving alternative transport options such as railroad infrastructures or by changing the values perceived by individuals (Meurs and Haaijers 2001; Taylor et al. 2008). Depending on how practical and affordable the various options are, individuals might be more or less likely to invest in an expensive car. Mobility options might influence the way households decide to invest their resources. Besides economic reasons, cultural factors might also affect the extent to which cars matter to individuals. The prevalence of materialistic or post-materialistic values may influence the relation between the life satisfaction of individuals and their cars.^1^ Previous research has indeed shown that Germans who follow materialistic “recipes” in their life choices score lower in their life satisfaction (Headey and Wagner 2019). In that sense, national conditions can influence the extent to which individuals invest and enjoy the functional and positional value of their cars.

This paper fills several gaps in the current literature. Although there is limited evidence on the links between cars and SWB, to the best of our knowledge, these links have not been observed from a cross-national perspective. Moreover, no study has investigated the role of contextual factors on this relationship. Therefore, the objective of the paper is twofold: First, we assess the importance of cars across different European countries especially through its functional and positional value on SWB. Second, we investigate why cars might be related to happiness in Europe through economic, infrastructural, and cultural factors. We do not look specifically at car ownership because this would lead us to other explanations (health, mobility, social capital…) that have been studied more intensively in previous literature especially since we are studying a senior population. In this paper, we focus on the influence of the value of cars on SWB.

This study is based on the Survey of Health, Aging, and Retirement in Europe (SHARE). Multilevel and fixed-effects regression models are used to analyze how cars, among other wealth components, are related to individuals’ life satisfaction in 18 European countries. This paper is organized as follows: the “Data and Methods” section presents the data and the methods used, and the “Results” section contains the results. The “Discussion” section discusses these results, and the “Conclusion and Limitations” section presents the conclusions.

## Data and Methods

We use SHARE data, which is an international, representative panel study of individuals whose age is at least 50 years old. Answers to questions on health, socio-economic status, social, and family networks are collected through computer-assisted personal interviewing (CAPI). A set of questions on the socio-economic status of respondents contains detailed self-reported information on household wealth under the form of assets and liabilities. The question on cars asks for the current market value of cars: “How much money would you get if you sold your car(s)?” This question does not give information about the purchasing price of cars because their value depreciates quite quickly, but gives a good picture of the value of these cars in terms of liquefiable wealth. Recent research shows that estimations made by individuals about their wealth possessions such as housing wealth are on average quite close to the estimates given by national accounts (Ravazzini et al. 2019). Cars are likely to be better evaluated than housing wealth because cars are more often replaced than houses. Furthermore, elderly people are likely to have purchased and sold several cars in their life acquiring some experience on how to evaluate the current market value of their cars during this process. There is currently no survey including all wealth components and questions on subjective well-being concurrently. In our case, despite not estimating consumer goods, SHARE includes one specific question about cars. Like all other wealth components, the value of cars has been imputed by the data provider. We take the five imputed values to perform the subsequent models.

Across the first six waves of SHARE, 21 countries participated at least once. We exclude wave 1 from the analysis because the question and the scale used to measure life satisfaction are different from the other waves. The question on SWB measures life satisfaction and asks: “how satisfied are you with your life as a whole?”. Answers are coded on an 11-point scale from 0 to 10. Apart from wave 1, we also exclude wave 3 because it focuses on people’s life stories, and it does not report quantitative information on wealth or life satisfaction. In all other waves, i.e., those numerated 2, 4, 5, and 6, SHARE collects information on household net worth concurrently as well as its components and life satisfaction. The main advantage of this database for our analysis is that the current market value of cars is clearly identified and is presented separately from other forms of real assets. Excluding two waves from the sample, the final sample used for the analysis contains 18 countries that participated in the survey at least twice and whose latest data is in wave 6 (2015) (or at least in wave 5) (2013).

The analysis is divided into two parts: The first part of the analysis identifies the effect of positive cars on subjective well-being first on average in all countries and then in each country. We restrict the sample to car owners to disentangle the effect of having an expensive car from the effect of having a car or not. The value of cars is measured at the household level. Therefore, we use multiple-level models in which individuals are clustered in households; for the average effect, households are also clustered in countries. After preliminary checks, we set gender as a random parameter because the effect on SWB is strongly country-dependent. Among all the variables we introduce, gender is the only parameter that can be set as random. We perform these multilevel (linear mixed-effects) models on the last available wave. We use a measure of wealth per capita because different household members could pool their resources, and, as in the case of cars, they might also use assets jointly.^2^ To do so, we divide wealth by the number of household members. Moreover, since respondents are 50 or older, the children that live in the household are likely to be old enough to drive. Due to the skewness, a transformation is necessary to normalize the distribution. We apply a commonly used transformation—inverse hyperbolic sine (IHS) transformation (see Pence 2006)—to cars to make it comparable to all other wealth components. Other wealth components need this transformation more than cars because of their high number of zeros and negative values. Even though this transformation has many important statistical properties, it also makes it difficult to interpret the size of the coefficients. To overcome this issue, we also discuss the coefficients obtained with untransformed wealth. In this case, the comparison of the coefficients associated with other wealth components is limited by the different skewness and number of 0s of each wealth component.

Apart from household wealth other than cars, a series of control variables are also included in the regression to account for possible confounders between the IHS-transformed wealth per capita and SWB. At the individual level, these variables include the age and the education of the respondents, gender, self-reported health status, marital status, household size, number of children in the household, his-transformed total household income, and type of area where the respondent lives (a big or a small city versus a rural area).

At the macro level, we also control for differences in national economic prosperity, which from now on we call economic “affluence” by including the logarithm of the GDP per capita provided by Eurostat. We perform regressions on absolute value of the car and on relative value of the car. In order to have information on peoples’ relative position of cars vis-à-vis others, we use average cars of the country, and we create a dummy variable for being among the top 25% of cars in each country. The top 25% corresponds to the first two top category used by Okulicz-Kozaryn et al. (2015) to define very good cars ($23,000–35,000) and luxury cars (> $35,000).^3^ In the first part of the analysis, we test whether cars have an impact on SWB. We also perform panel fixed-effect regressions on all years and all countries of the sample to test whether a change in the value of cars is associated with a change in SWB. We use all individual variables presented before, except for gender and education, which remain unchanged over time. We perform likelihood ratio tests to observe whether certain factors introduce random effects.

In the second part of the analysis, we investigate which contextual variables mediate the effect of car value on SWB. We investigate economic, infrastructural, and cultural factors. These variables are represented by the GDP, the quality of train infrastructure collected by the World Economic Forum (WEF) for the Global Competitiveness Report (Schwab and Sala-i-Martin 2015), and materialistic values as measured by Schwartz (2012) in the European Social Survey. The quality of train infrastructures is measured via an expert opinion using a scale that goes from 1 (extremely underdeveloped) to 7 (extensive and efficient). This indicator is currently used by the European Commission in the scoreboard to measure the quality of transports in the European Union (EC 2018). Materialistic values and the importance of being recognized as successful are measured among other values in the European Social Survey using the values of Schwartz (2012). Two items are particularly interesting in our case: “important to be rich, have money and expensive things” and “important to be successful and that people recognize achievements.” The answers follow a scale that goes from 1 “Not like me at all” to 6 “Very much like me”.^4^ We match the average values among 50+ in each country pairing the rounds of the ESS to the waves of SHARE. If the exact round is not available, then we take information from the closest available round.^5^ To test the influence of contextual variables on the role of cars, GDP, the quality of railroad infrastructures, and values are interacted with car value in multilevel regressions. Each variable is tested separately because the multilevel models run on 18 countries and do not support more than one interaction with a variable at the macro level. With our database, the concurrent inclusion of contextual variables is not possible; therefore, the shared variance among the contextual variables is not considered in the models. This is a limitation of our study, which might be solved in the future with a larger database of countries. The contextual variables are also not tested in the current longitudinal models because, apart from the GDP, the other variables have either methodological inconsistencies (the quality of train infrastructure) or do not have sufficient year combinations per country (values).

## Results

The average relationship between the value of the car and SWB is illustrated in Table 1. The coefficients show the influence of different variables (value of the cars per-capita, average value of cars of the country, and the fact of being in the top 25% of the values of cars together with other wealth components and GDP per capita) on SWB. Individual controls are included but are not displayed for brevity. In model 1, the value of cars per-capita is presented together with other wealth components that are then aggregated from model 2 onwards. To observe the effect of relative value of cars on SWB, models 3 and 4 introduce the average value of cars and the fact of being in the top 25% of value of cars, respectively.

**Table 1:** The relationship between the value of cars and life satisfaction; cross-sectional evidence

	(1)	(2)	(3)	(4)
	Life satisfaction	Life satisfaction	Life satisfaction	Life satisfaction
Car value	0.104***	0.113***	0.113***	0.104***
	(7.97)	(7.97)	(7.65)	(7.10)
Average car value			0.034	
			(0.337)	
Top 25% car value				0.038
				(1.48)
Primary home wealth	0.010***			
	(3.83)			
Other real estate	0.004***			
	(2.99)			
Bank accounts	0.028***			
	(6.46)			
Bonds, stocks and mutual funds	0.004**			
	(1.99)			
Savings for long-term investments	0.010***			
	(5.64)			
Value of business	0.001			
	(0.30)			
Liabilities	− 0.025***			
	(− 4.11)			
Mortgages	− 0.007***			
	(− 4.25)			
Other wealth		0.034***	0.034***	0.034***
		(6.79)	(6.79)	(6.79)
Income	0.021*	0.027**	0.027**	0.027**
	(1.67)	(2.32)	(2.67)	(2.310)
GDP	0.255**	0.269**	0.270*	0.279**
	(2.25)	(2.28)	(2.16)	(2.21)
Var. (gender)	.0556632	.0044005	.0044075	.0043926
Var. (constant)	.0044448	.060599	.0604622	.0607226
Var. (residuals)	1.99	1.42	1.42	1.42
*N* of countries	18	18	18	18
*N* of observations	249,740	249,740	249,740	249,740

Sources: SHARE W6 for individual variables in 16 countries, SHARE W5 for individual variables for the Netherlands and Israel, Eurostat for the GDP per capita. Notes: additional controls (age, education, gender, household size, number of children, marital status, the urbanization of the place of residence, and subjective health) are included in all models but are not displayed for brevity. Wealth components and income are transformed with an inverse hyperbolic sine transformation, and GDP is transformed with the logarithm

*T* statistics are reported in parenthesis. ***, **, and * indicate significance at the 1%, 5%, and 10% level respectively

**Fig. 1:**
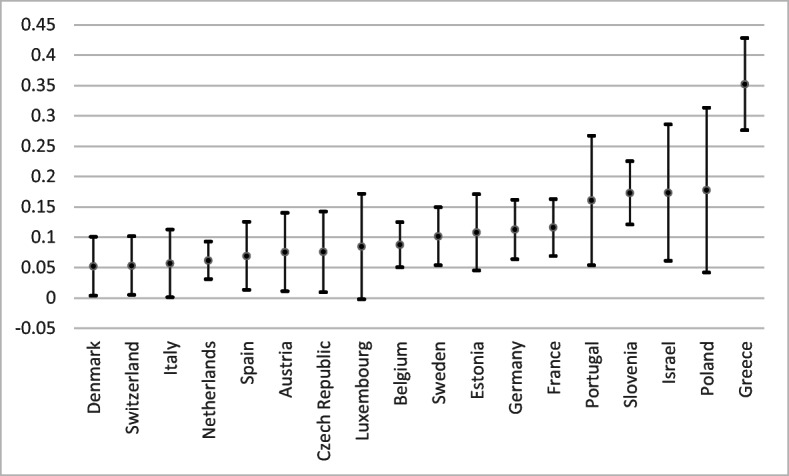
Country-specific effects of car value on life satisfaction across 18 countries. (The figure provides the effects of car wealth on SWB. They are ranked from the country where the effect is the lowest (Denmark) to the country where the effect is the largest (Greece). ) Sources: SHARE W6 for 16 countries; SHARE W5 for the Netherlands and Israel. Notes: Bars indicate 95% confidence intervals. Wealth transformed according to an inverse hyperbolic sine transformation

The coefficient of car value indicates a positive and significant relationship with SWB (model 1). This means that, as far as European seniors are concerned, expensive cars increase life satisfaction more than economical cars.^6^ In model 1, the effect of car value on SWB is positive and significant, and is higher than the effect of income and the other wealth components on SWB available in SHARE. This holds when all wealth components are taken separately and when they are combined as shown in model 2. Even though these coefficients are based on a cross-sectional analysis, we suggest that an increase of car value of 1000 EUR is associated with an increase of SWB of 0.020 points on a scale from 0 to 10 (see Table 8 in the Appendix). Put differently, if my neighbor has a car that is 10,000€ more expensive than mine (around the average), he or she should enjoy a surplus of 0.20 point of life satisfaction compared to me all other things being equal. The effect of car value is also the strongest in longitudinal analyses (see models 3 and 4 in Table 8). The coefficients for car value in models 1 and 2 in Table 1 show the average relationship between car value and SWB in all 18 countries used for the analysis. However, it is likely that the relationship differs according to the country. Figure 1 confirms this possibility. The weakest effect is in Denmark, and the strongest effect is in Greece. While the size effects vary from one country to another, the relationship between car value and life satisfaction is positive and significant in every country of the study.

After assessing the importance of the value of cars, we now study the positional effect of cars. Models 3 and 4 in Table 1 show that the positional value of cars is not so important for SWB. Controlling for the GDP, we find that SWB is not significantly related with the average value of cars in a country. This evidence agrees with Okulicz-Kozaryn et al. (2015). Since there are different relationships among countries, we continue the analysis by assessing the possible moderating effect of the country-specific contexts in the relationship between cars and SWB. We do so by regressing car value together with economic, infrastructural, and cultural factors. Interactions include the level of affluence of a country (model I1), the quality of trains (model I2), the importance of being rich (model I3), and the importance of being successful (model I4). The results are presented in Table 2.

**Table 2:** The influence of contextual variables on the effect of the functional and the positional value of cars on SWB

	(I1)	(I2)	(I3)^a^	(I4)^b^
	Life satisfaction	Life satisfaction	Life satisfaction	Life satisfaction
Car value	0.987***	0.306***	− 0.045	0.019
	(3.34)	(4.76)	(− 0.28)	(0.17)
Car value & GDP	− 0.085***			
	(− 3.04)			
Quality of trains		0.381**		
		(2.25)		
Car value & quality of trains		− 0.041***		
		(− 3.18)		
Importance of being rich			− 0.385	
			(− 0.55)	
Car value & being rich			0.061	
			(0.94)	
Importance of being successful				− 0.10
				(− 0.28)
Car value & being successful				0.025
				(0.85)
Other wealth	0.033***	0.033***	0.033***	0.033***
	(6.65)	(6.65)	(6.53)	(6.56)
GDP	0.929***	0.172	0.295***	0.240**
	(2.98)	(1.17)	(2.15)	(1.94)
Constant	− 6.14* (− 1.89)	− 0.076 (− 0.11)	1.82 (0.91)	1.31 (0.64)
Var. (gender)	.0043264	.0044678	.0044534	.0044965
Var. (constant)	.0638549	.0586227	.0598053	.0575489
Var. (residuals)	2.004969	2.004824	2.006668	2.006919
N of countries	18	18	18	18
N of observations	244,675	244,675	244,675	244,675

Sources: SHARE W6 for individual variables in 16 countries; SHARE W5 for individual variables for the Netherlands and Israel; Eurostat for the GDP per capita; Global Competitiveness Report of the World Economic Forum for the quality of train infrastructure; and the ESS for the importance of being rich or successful. Notes: additional controls (age, education, gender, household size, number of children, marital status, income and the urbanization of the place of residence, and subjective health) are included in all models but not displayed for brevity. Wealth components and total household income are transformed with an inverse hyperbolic sine transformation and GDP is transformed with the logarithm

*T* statistics are reported in parenthesis. Symbols ***, **, and * indicate significance at the 1%, 5%, and 10% level respectively

^a^Without the interaction term, the effects of car value is 0.113*** and importance of being rich 0.5 (ns)

^b^Without the interaction term, the effects of car value is 0.113*** and importance of being successful 0.8 (ns)

The introduction of an interaction with the level of affluence affects the influence of cars on SWB (I1). The negative interaction term indicates that the relation between cars and SWB is less important in more affluent nations. Regarding the level of infrastructure, the quality of trains has a positive impact on people’s SWB (model I2). The significance of the interaction term between car value and quality of trains shows that higher train quality decreases the impact of car value on SWB. In addition, the loss of the significance level of the GDP indicates that the quality of trains accounts for the general level of affluence in the country. In models I3 and I4, the main effects are no longer significant when including the interaction term between car value and importance of being rich and car value and the importance of being successful even though the interaction between the importance of being rich and car value is not significant. Overall, the variance at the individual level is, however, much higher than the variance at the country level (ICC = 0.03). This means that individual characteristics are the most important source of variability in the relationship between cars and SWB.

We also test these relationships with fixed-effect models. The coefficient of car value is still positive and significant meaning that positive changes in car value at the individual level are associated with positive changes of life satisfaction (Table 3). The relationship is significant only for some other wealth components—namely primary home wealth, bank accounts, savings for long-term investments and liabilities. Car value remains the highest coefficient in magnitude among all wealth components. Comparing L2 and model 2, the longitudinal effect of car value is lower than the cross-sectional effect. This also holds in the case of untransformed coefficients of car value, i.e., an increase of 1000 EUR in car value augments SWB by 0.004 points versus the 0.020 points found in the cross-sectional analysis (Table 8). This means that part of the coefficients in Table 1 is explained by unobservable characteristics and national conditions. Still, the coefficient in the longitudinal regression remains positive and significant. The relationship of cars to SWB is moderated by the level of affluence of the country (L4). If the level of affluence of a country increases, then cars become less important for SWB. These longitudinal models explain 60% of the time variance in SWB.

**Table 3:** The relationship between car value and life satisfaction (longitudinal evidence)

	(L1)	(L2)	(L3)	(L4)
	Life satisfaction	Life satisfaction	Life satisfaction	Life satisfaction
Car value	0.031***	0.034***	0.031***	0.550***
	(5.074)	(6.627)	(4.855)	(4.913)
Primary home wealth	0.003*			
	(1.386)			
Other real estate	− 0.001			
	(− 0.814)			
Bank accounts	0.008***			
	(3.401)			
Bonds, stocks and mutual funds	0.002			
	(1.042)			
Savings for long-term investments	0.004***			
	(2.354)			
Value of business	0.002			
	(0.620)			
Liabilities	− 0.005***			
	(− 2.759)			
Mortgages	− 0.001			
	(− 0.463)			
Top 25% car wealth			0.013	
			(0.884)	
Car value & GDP				− 0.050***
				(− 4.620)
Other wealth		0.010***	0.010***	0.010***
		(5.930)	(5.380)	(5.362)
GDP	0.519***	0.119	0.122	0.521***
	(3.468)	(1.659)	(1.545)	(4.431)
R2	0.60	0.60	0.60	0.60
N of observations	772,405	772,405	772,405	772,405
N of countries	18	18	18	18

Sources: SHARE W2-6 for individual variables in 18 countries; Eurostat for the GDP per capita. Notes: additional controls (age, household size, number of children, marital status, the urbanization of the place of residence and subjective health) are included in all models but not displayed for brevity. Wealth components and total household income are transformed with an inverse hyperbolic sine transformation, and GDP is transformed with the logarithm

*T* statistics are reported in parenthesis. ***, **, and * indicate significance at the 1%, 5%, and 10% level, respectively

## Discussion

We found that cars have a significant and positive influence on the happiness of seniors in Europe. Their contribution is higher than housing wealth, money available for current expenditures, and other financial assets. This relationship between cars and SWB is observable in every European nation at different intensities. Differences across countries are linked to economic, infrastructural, and cultural factors. The relationship is also observed in longitudinal models. This increase is moderated by the level of affluence of the country: If there is economic growth, then an increase in car value becomes less important for SWB.

The main results are summarized in Table 4 here below.

**Table 4:** Main results

	Results
Direction and intensity of the relation	The relation between cars and SWB exists, and it is positive and significant in 18 European countries. The effect is stronger in Greece and weaker in Denmark. An increase of 1000 EUR in car value augments SWB by 0.020 points in cross-sectional analyses, 0.004 in longitudinal analyses.
Moderators at the macro level: economic aspects	The relation between SWB and the interaction term between GDP and car value is significant and negative. There is a moderating effect of economic prosperity.
Moderators at the macro level: infrastructural aspects	The relation between SWB and the interaction term between quality of infrastructures and car value is negative and significant. There is a moderating effect of the quality of infrastructures.
Moderators at the macro level: values	The main effect disappears when introducing the interaction terms SWB and “importance of being rich” and “importance of being successful.” There is a moderating effect of materialistic values.

### The Influence of the Context on the Relation Between Cars and SWB

One of the main findings of this paper is that the influence of cars matters more in less affluent countries although the relation is far from being systematic. Although seniors in countries such as Denmark enjoy having a more expensive car, they do so to a lesser extent than seniors in less affluent countries such as Greece. Part of this can be explained by the quality of infrastructures especially train infrastructure. Cars matter less in places where train infrastructures are better. Besides economic and infrastructural reasons, one can also see that values have an influence on the car-SWB relation. In countries where material possessions are more valued, owning an expensive car brings more happiness. Once introduced, the interaction term between importance of being successful and car value as well as the interaction term between importance of being rich and car value make the car-SWB relation insignificant. This also means part of the relation is carried out by values.

Contrary to the contribution of Okulicz-Kozaryn et al. (2015) in the USA, it seems that in Europe, having a more expensive car than an average frugal car is influential for seniors’ SWB. However, there is a difference between the two studies in the population studied; Okulicz-Kozaryn et al. (2015) observed the entire population while we only examined seniors. Materialistic values are more prominent among older generations (Inglehart 2008); this is likely to have an influence. Furthermore, public transportation is more developed in Europe than in the USA, and the effect of cars in Europe is probably more positional because the functional value can be derived by other means of transportation.

### Functional Value or Positional Value

Hirsch (1976) distinguishes the functional value from the positional value of goods in general. We defined the functional value of cars as a driving unit and the positional value as anything that would come on top. Thus defined, this means that households owning a car can benefit from the functional value of the car, and the positional value is represented by the relative value of the car. In this case, a more expensive car has a higher positional value than a cheaper one independent of the type of car other people own. Our analysis shows that both the functional and positional values of a car have independent and additive effects on the life satisfaction of seniors. Individuals benefit from having a car, and more expensive cars bring more satisfaction. Here, there are two ways in which we operationalized the positional value of cars: (1) by looking at the national average car value and 2) by looking at the top 25% of car value in each country. These values are considered to be average and do not include the individual passion for driving that some people would consider as pleasure and is not necessarily positional. Furthermore, functional and positional values might not be fixed concepts; functional and positional value could evolve with economic growth or technological evolutions. What used to once be considered functional and positional values might become simply functional after some time once the item is considered to be “normal” (e.g., air conditioning in European countries).

## Conclusion and Limitations

Cars are the most significant wealth component among all wealth components measured by SHARE. Car value is positively related to seniors’ SWB in the 18 European countries analyzed here. This relation is true in a static picture—at a given point, controlling for income and other wealth sources, individuals with high car value are happier. The relationship is also observable in a dynamic picture when given individuals become happier when they acquire more car value. The relationship in a static picture suggests that 1000 EUR more in car value is associated with a SWB that is 0.020 points higher. The relationship in a dynamic picture indicates that an increase of 1000 EUR of car value augments SWB by 0.004 points. The impact on SWB is not very high in either case among all wealth components. This relative component of cars is more important in some countries like Austria, Germany, the Netherlands, and Italy than in other European countries. In general, cars are also more important for SWB in Greece than in Denmark or in Switzerland. The difference in the relationship between cars and SWB across Europe can be at least partly explained by macro factors. The importance of cars is lower where economic affluence is higher, where train infrastructures are better, and where post-materialistic values prevail.

This result could be relevant in a context where government develop sustainable development policies. They sometimes do so by imposing (eco)taxes on cars with high carbon emissions. The rationale is that car consumption would go down by increasing the prices paid by car consumers. However, it is possible that, instead of discouraging consumption, these policies could also increase car consumption making people willing to pay more to increase their SWB. If that was the case, then this would be particularly true in settings where materialistic values are prevalent. Thus, one needs to understand the effect of the functional and positional value of cars on SWB because it can explain how to act efficiently to avoid the increase of car uses resulting in undesired consequences such as pollution and congestion (in a “tragedy of the commons” type of scenario). Although a positive link between cars and seniors’ SWB goes against sustainable development, this result must be considered if one wants to resorb the environmental Attitude-Behavior Gap that represents the tendency of people to report more green attitudes and to act in an unsustainable manner.

The relevance of the economic affluence and infrastructural development of the country also suggests that cars probably become less important for SWB of the elderly through economic growth and investments in public transport. The importance of cars might also change with cohort replacement to alter the dominant materialistic values among European countries. Future studies might focus more on the psychological mechanisms and the micro determinants that link SWB to cars. Future research is also needed to identify the relationship between cars and SWB among other age groups.

## Figures and Tables

**Table 6:**
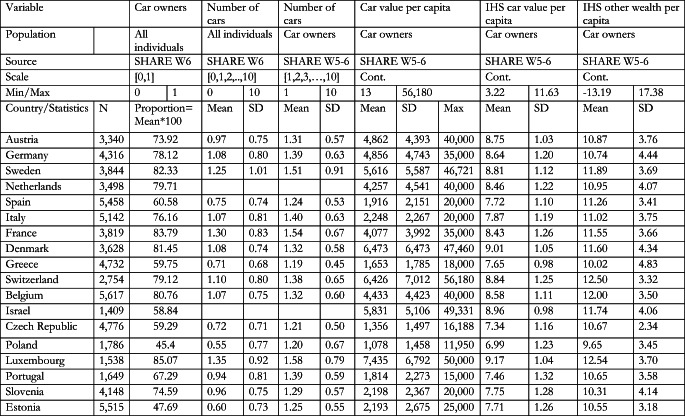
Descriptive statistics of car owners, the number of cars, and car value

Notes: Wave 5 is used only for the Netherlands and Israel

## Appendix

**Table 5:** Effect of car ownership, the number of cars, and car value on life satisfaction

	(1)	(2)	(3)	(4)	(5)	(6)
	Lifesatisfaction	Lifesatisfaction	Lifesatisfaction	Lifesatisfaction	Lifesatisfaction	Lifesatisfaction
Car owner	0.264***					
	(8.661)					
No car (ref.)						
1 car		0.255***				
		(8.697)				
2 cars		0.382***				
		(7.366)				
3 cars		0.376***				
		(6.098)				
4 cars		0.470***				
		(5.568)				
5+ cars		0.136				
		(1.111)				
Car value per car				0.100***		
				(6.242)		
Car value					0.104***	0.107***
					(6.629)	(7.168)
Number of cars					0.025**	
					(2.224)	
Age	0.017***	0.018***	0.018***	0.016***	0.017***	0.016***
	(12.962)	(13.025)	(12.589)	(14.249)	(14.810)	(14.418)
Gender (f)	0.034	0.036	0.039*	0.023	0.028	0.027
	(1.470)	(1.597)	(1.692)	(1.219)	(1.450)	(1.393)
Household size	0.025***	0.019**	0.041***	0.061***	0.054***	0.058***
	(2.792)	(2.252)	(4.079)	(5.667)	(5.200)	(5.626)
Number of children	0.033***	0.034***	0.036***	0.032***	0.032***	0.033***
	(4.984)	(5.191)	(5.681)	(4.454)	(4.608)	(4.632)
Income	0.039***	0.037***	0.034***	0.030**	0.027**	0.028**
	(3.648)	(3.556)	(3.268)	(2.413)	(2.232)	(2.248)
Married (ref.)						
Single	− 0.283***	− 0.268***	− 0.268***	− 0.285***	− 0.270***	− 0.273***
	(− 8.165)	(− 7.797)	(− 7.564)	(− 6.218)	(− 5.941)	(− 5.913)
Separated	− 0.359***	− 0.348***	− 0.349***	− 0.319***	− 0.308***	− 0.310***
	(− 10.720)	(− 11.187)	(− 10.740)	(− 9.172)	(− 9.359)	(− 9.204)
Widowed	− 0.280***	− 0.278***	− 0.281***	− 0.385***	− 0.381***	− 0.382***
	(− 7.492)	(− 7.607)	(− 7.659)	(− 9.265)	(− 9.098)	(− 9.145)
Compulsory education (ref.)						
Lower secondary	0.033	0.030	0.026	0.021	0.017	0.018
	(0.833)	(0.765)	(0.647)	(0.534)	(0.448)	(0.459)
Upper secondary	0.069**	0.062*	0.056*	0.032	0.023	0.025
	(2.064)	(1.870)	(1.718)	(1.173)	(0.870)	(0.917)
Tertiary	0.098*	0.084*	0.074	0.056	0.041	0.044
	(1.869)	(1.676)	(1.562)	(1.131)	(0.863)	(0.914)
Working	0.041**	0.037**	0.035**	0.022	0.018	0.019
	(2.391)	(2.147)	(2.075)	(1.551)	(1.215)	(1.287)
Urban area	− 0.105***	− 0.098***	− 0.103***	− 0.102***	− 0.095***	− 0.097***
	(− 6.067)	(− 5.792)	(− 5.931)	(− 5.506)	(− 5.144)	(− 5.215)
Other wealth	0.034***	0.033***	0.032***	0.036***	0.035***	0.035***
	(6.346)	(6.319)	(6.215)	(5.847)	(5.710)	(5.718)
GDP	0.378***	0.365***	0.391***	0.283**	0.268**	0.269**
	(3.155)	(3.017)	(3.224)	(2.326)	(2.169)	(2.186)
Subjective health	0.499***	0.498***	0.493***	0.438***	0.436***	0.436***
	(24.321)	(24.488)	(24.468)	(27.558)	(27.849)	(27.746)
Constant	0.285	0.388	0.108	0.999	1.062	1.060
	(0.234)	(0.315)	(0.086)	(0.790)	(0.831)	(0.832)
*N* of countries	16	16	16	16	16	16
*N* of observations	63,865	63,865	63,865	45,827	45,827	45,827

Sources: SHARE W6 for individual variables in 16 countries; Eurostat for the GDP per capita. Regressions 4, 5, and 6 are restricted to car owners

***, **, and * indicate significance at the 1%, 5% and 10% level, respectively

**Table 7:** Descriptive statistics of macro variables for the last available wave

Variable	Quality of trains	Quality of roads	Log. GDP per capita	Importance of being rich	Importance of being successful
Source	GCR	GCR	Eurostat	ESS	ESS
Scale	Cont.	Cont.	Cont.	Cont.	Cont.
Country/Statistics	Mean	Mean	Mean	Mean	Mean
Austria	5.32	5.99	10.59	2.71	3.84
Germany	5.36	5.55	10.53	2.33	3.71
Sweden	4.11	5.29	10.73	2.28	2.80
Netherlands	5.59	6.14	10.57	2.53	3.61
Spain	5.62	5.52	10.06	2.46	3.28
Italy	4.11	4.55	10.21	2.86	4.23
France	5.83	6.05	10.40	2.10	2.77
Denmark	4.7	5.71	10.77	2.61	3.59
Greece	2.84	4.3	9.69	3.25	3.25
Switzerland	6.6	5.9	11.21	2.50	3.80
Belgium	4.88	4.88	10.51	2.49	3.61
Israel	3.2	5	10.23	3.18	4.39
Czech Republic	4.55	4.1	9.68	3.25	3.64
Poland	3.34	3.97	9.32	2.95	3.56
Luxembourg	5.06	5.57	11.43	2.59	3.43
Portugal	4.16	5.91	9.76	2.07	3.74
Slovenia	2.97	4.42	9.84	2.60	4.39
Estonia	4.02	4.67	9.65	2.61	3.34

**Table 8:** Effect of untransformed car value on life satisfaction

	(1)	(2)	(3)	(4)
	Cross-sectional	Cross-sectional	Longitudinal	Longitudinal
Car value	0.020***	0.021***	0.004***	0.004***
	(5.09)	(5.09)	(2.469)	(3.470)
Primary home wealth	0.000**		0.000	
	(2.39)		(0.200)	
Other real estate	0.000***		0.000	
	(2.51)		(0.665)	
Bank accounts	0.001***		0.000**	
	(3.84)		(0.625)	
Bonds, stocks and mutual funds	0.000		0.000	
	(1.06)		(1.17)	
Savings for long-term investments	0.000*		0.000	
	(1.95)		(.32)	
Value of business	− 0.000		0.000	
	(− 1.28)		(0.451)	
Liabilities	− 0.003***		0.000	
	(− 2.61)		(0.553)	
Mortgages	− 0.001**		0.000	
	(− 2.10)		(−0.01)	
Other wealth		0.000***		0.000*
		(3.22)		(2.103)
GDP	0.310***	0.320***	0.52***	0.141*
	(2.46)	(2.58)	(3.58)	(1.949)
Var (gender)	.0045342	.0044786		
Var (constant)	.0580759	.0563713		
Var (residuals)	2.019885	2.022639		
*R* squared	–	–	0.60	0.60
*N* of countries	18	18	18	18
*N* of observations	49,548	49,548	131,312	154,509

Sources: SHARE W6 for individual variables in 16 countries; SHARE W5 for individual variables for the Netherlands and Israel; Eurostat for the GDP per capita. Notes: additional controls (age, education, gender, household size, number of children, marital status, the urbanization of the place of residence, and subjective health) are included in all models but not displayed for brevity. All wealth components and total household income are untransformed but divided by 1000 to reduce the number of decimals. GDP is transformed with the logarithm

*T* statistics are reported in parenthesis. ***, **, and * indicate significance at the 1%, 5%, and 10% level, respectively

## Footnotes

1 Feminine and masculine values can be associated to materialistic values. Campbell (1995) shows, for instance, that the boom of the SUV in the USA is linked to masculine and military values following the geopolitical insecurity of the early 2000s.

2 The highest car wealth dividing by the household size is 56,180 euros in Switzerland and not dividing by the household size it becomes 70,000 euros in Luxembourg. The number of cars is available only in wave 6. In this wave, we perform additional tests of the relationship between car wealth and SWB controlling for car ownership, the numbers of cars, and dividing car wealth per capita by the number of cars.

3 Not in all countries, the top category has more than $35,000 in car wealth (see the car maximum wealth per capita in Table 6 in the Appendix). The correspondence with Okulicz-Kozaryn et al. (2015) paper is in terms of the distribution than in terms of absolute values.

4 The original scale of the ESS has been reversed to have larger scores meaning a higher correspondence to the indicated value.

5 This concerns Greece, Italy, and Luxembourg.

6 Results in the Appendix (Table 5) illustrate that owning a car is associated with higher levels of satisfaction versus not owning a car. Looking at car value only but not controlling for the number of cars (M6) gives a very similar coefficient (0.107). This suggests a greater importance of the value of cars than in the number of cars. Descriptive statistics of the number of cars, car wealth, and other wealth—as well as macro variables—are presented in Table 6.
